# Risk of major adverse events associated with gabapentinoid and opioid combination therapy: A systematic review and meta-analysis

**DOI:** 10.3389/fphar.2022.1009950

**Published:** 2022-10-11

**Authors:** Jongsung Hahn, Youngkwon Jo, So Hee Yoo, Jaekyu Shin, Yun Mi Yu, Young-Mi Ah

**Affiliations:** ^1^ School of Pharmacy, Jeonbuk National University, Jeonju, Jeonbuk, South Korea; ^2^ KIURI Research Center, Ajou University, Suwon, South Korea; ^3^ Department of Pharmacy and Yonsei Institute of Pharmaceutical Sciences, College of Pharmacy, Yonsei University, Incheon, South Korea; ^4^ Department of Clinical Pharmacy, School of Pharmacy, University of California, San Francisco, San Francisco, CA, United States; ^5^ Department of Pharmaceutical Medicine and Regulatory Sciences, Colleges of Medicine and Pharmacy, Yonsei University, Incheon, South Korea; ^6^ College of Pharmacy, Yeungnam University, Gyeongsan, Gyeongsangbuk-do, South Korea

**Keywords:** opioid, gabapentin, pregabalin, safety, mortality

## Abstract

**Background:** The use of opioid–gabapentinoid combinations has increased, raising several safety concerns. However, meta-analysis studies focusing on this issue are limited.

**Objective:** To evaluate the risk of central nervous system (CNS) depression, gastrointestinal (GI) adverse events, and mortality of combination therapy compared with those of opioid therapy and to explore the differences in the results according to study design and indications.

**Methods:** Relevant studies were selected (published before 30 January 2022) by searching the MEDLINE, Embase, and CENTRAL databases. The pooled odds ratios (OR) with 95% confidence intervals (CI) of the outcomes were estimated using the Mantel–Haenszel method. Subgroup and meta-regression analyses were performed according to study characteristics. Quality assessment was conducted using the Risk of Bias 2 tool for randomized controlled trials (RCTs) and Cochrane Collaboration’s Risk of Bias in non-RCTs tool for non-randomized trials.

**Results:** Adverse events were reported in 26 RCTs and 7 non-RCTs, and mortality was reported in 10 non-RCTs. Compared to opioid therapy, dizziness, cognitive dysfunction, and respiratory depression in combination therapy significantly increased in non-RCTs (OR 3.26, 95% CI 1.82–5.85; OR 3.13, 95% CI 1.51–6.50; OR 1.71, 95% CI 1.31–2.24, respectively), and a similar trend for dizziness and cognitive dysfunction was also identified in the RCT analysis, although the difference was not significant. Combination therapy for cancer pain was associated with the highest risk of sedation in subgroup analysis. Combination therapy significantly decreased the risk of GI adverse events, including nausea, vomiting, and constipation. The mortality risk associated with combination therapy was higher than that associated with opioid therapy (OR 2.76, 95% CI 1.26–6.05).

**Conclusion:** Opioid-gabapentinoid combination therapy could be associated with an increased risk of CNS depression and mortality, despite tolerable GI adverse events. These data suggest that combination therapy requires close monitoring of CNS depression, especially in cancer patients. Caution is needed in interpreting the clinical meanings owing to the lack of risk difference in respiratory depression in the RCT-only analysis and the absence of RCT or prospective studies investigating mortality.

## 1 Introduction

Opioid therapy is a major treatment for moderate-to-severe pain associated with surgery, injury, or cancer. However, with the increasing opioid overdoses and opioid-related deaths (Scholl et al., 2018; CDC, 2019), multimodal analgesia involving opioids and non-opioid analgesics with different mechanisms of action has emerged as a strategy to reduce reliance on opioids and effectively control pain (Dowell et al., 2016; Ramirez et al., 2020).

Gabapentin and pregabalin, jointly referred to as gabapentinoids, are commonly used nonopioid analgesics. They are used to treat diabetic neuropathy, fibromyalgia, and postherpetic neuralgia (Goodman and Brett, 2017; Montastruc et al., 2018). In 2017, more than 20% of patients in the United Kingdom who were newly prescribed gabapentinoids were taking opioids concomitantly (Montastruc et al., 2018). In the United States, prescriptions of gabapentinoids increased by about 50% between 2012 and 2016 (Goodman and Brett, 2017).

Gabapentinoids have some safety concerns regarding central nervous system (CNS) depression in that they can cause sedation and dizziness and may lead to cognitive impairment in some patients (Goodman and Brett, 2017). Also, simultaneous use of gabapentinoids with an opioid may change the risk of adverse events associated with opioid use (Kardas et al., 2020). A recent meta-analysis showed that the perioperative therapy of administering a gabapentinoid with an opioid in patients with lower limb arthroplasty reduced the risk of postoperative nausea, vomiting, and pruritus, but not sedation (Campbell et al., 2021). This meta-analysis included only randomized controlled trial (RCT) studies that mostly focused on the short-term use of perioperative analgesics. However, gabapentinoids are prescribed for long-term use for cancer-associated or non-cancer chronic pain, and their medication use could be different from RCT studies in actual clinical settings (Chen et al., 2016; Yu et al., 2021).

The 2019 Beers Criteria recommend avoiding a combination of opioids and gabapentinoids owing to the potential risk of respiratory depression (AGS Beers Criteria Update Expert Panel, 2019). Furthermore, the concurrent use of opioids with gabapentinoids increased mortality risk as demonstrated in an analysis of death registration in the United Kingdom (Chen et al., 2022). However, to the best of our knowledge no meta-analysis has examined the mortality risk associated with the combined use of gabapentinoids and opioids. Therefore, to comprehensively evaluate the safety of gabapentinoids and opioid combinations, a multi-faceted evaluation considering the characteristics of medication use according to indications and real-world evidence is necessary.

This study performed a systematic review and meta-analysis to evaluate the risk of CNS depression, gastrointestinal (GI) adverse events, and mortality when gabapentinoids were used with opioids. Given the difference in study design between RCTs and non-RCTs, we explored the results according to the study design by considering clinical factors such as indications and intervention type.

## 2 Materials and methods

This study followed the guidelines recommended by the Preferred Reporting Items for Systematic Reviews and Meta-analyses 2020 (PRISMA 2020) (Supplementary Table S1) (Page et al., 2021). The study protocol is available in the PROSPERO database (CRD42022302896). Two investigators (YKJ and SHY) independently performed the literature search, study selection, data extraction, and quality assessment. Discrepancies, if any, were resolved by two other investigators (YMY and YA).

### 2.1 Search strategy

The MEDLINE, EMBASE, and CENTRAL electronic databases were systematically searched for relevant studies published before 30 January 2022. The search used a combination of medical subject headings and the keywords “opioid analgesics” and “gabapentinoids.” The complete search strategy used in this analysis is listed in Supplementary Table S2.

### 2.2 Study selection

Studies were considered eligible if they met the following inclusion criteria: 1) population: enrolled adult patients aged 18 years or older undergoing pain management; 2) intervention: a combination of opioid analgesics and gabapentinoids use for more than 24 h; 3) comparison: opioid analgesic use for more than 24 h; 4) outcomes: the risk of adverse events and death; and 5) study design: prospective or retrospective studies. The following studies were excluded: 1) non-human studies, including animal and *in vitro* studies; 2) reviews, meta-analyses, or ongoing studies; 3) case reports; 4) studies available only in the form of abstracts or posters; and 5) publications not in English.

### 2.3 Data extraction

Eligible studies were reviewed, and the following data were extracted using a standardized extraction form: first author, publication year, country, study design, database used in the study, number of patients, sex, age, indications, regimens of opioid analgesics and gabapentinoids, duration of treatment, duration of follow-up, and details of adverse events.

### 2.4 Study outcomes

The primary study outcomes were treatment-related adverse events (TRAEs) such as CNS depression and GI adverse events. CNS depression includes sedation, dizziness, cognitive dysfunction, and respiratory depression. GI adverse events included nausea, vomiting, and constipation. Mortality rate was also evaluated.

### 2.5 Analysis

In this study, we analyzed the risk of TRAEs and death according to the study design (i.e., RCTs and non-RCTs). The pooled odds ratios (ORs) with 95% confidence intervals (CIs) of TRAEs and deaths associated with the use of opioids and gabapentinoids were computed using the Mantel–Haenszel method. OR and hazard ratio (HR) data for mortality adjusted for confounding factors (such as sex, year, comorbid diseases, and concurrent medications) were weighted and pooled using the generic inverse-variance method. Heterogeneity was assessed using inconsistency statistics (*I* ^2^), with significance set at *I* ^2^ > 50% (Higgins and Thompson, 2002). A common-effects model was used in the absence of significant heterogeneity, and a random-effects model was employed when significant heterogeneity was present (Higgins et al., 2019).

We conducted subgroup and meta-regression analyses of RCTs. We evaluated differences in TRAEs between combination therapy and opioid therapy according to indications (perioperative pain, non-cancer chronic pain, and cancer-associated pain), duration of treatment, prescription dosage-morphine milligram equivalents (MME) of oral opioids and defined daily doses (DDDs) of gabapentinoids. Sensitivity analysis was conducted by removing low-quality studies or adding each study in the order of sample size to determine the robustness of the results.

Quality assessment of each included study was conducted using the Risk of Bias 2 (RoB 2) tool for RCTs (J. A. C. Sterne et al., 2019) and the Cochrane Collaboration’s Risk of Bias in non-RCTs (ROBINS-I) tool for non-randomized trials (J. A. Sterne et al., 2016). Publication bias was examined using funnel plots and Egger’s regression test. Statistical significance was defined as *p* < 0.05. The meta-module in R 4.1.0 (R Foundation for Statistical Computing, Vienna, Austria) was used for statistical analysis.

## 3 Results

### 3.1 Study selection

Supplementary Figure 1 shows the process of selecting eligible studies according to the PRISMA 2020 guidelines. After excluding duplicates, 3,699 articles were screened for relevance based on the title and abstract, and 3,520 articles were excluded. After 179 relevant articles were assessed for eligibility through a full-text evaluation, 43 studies with 6,537,444 patients were selected. TRAEs were reported in 26 RCTs (Caraceni et al., 2004; Gilron et al., 2005; Fassoulaki et al., 2006; Turan et al., 2006; Keskinbora et al., 2007; Clarke et al., 2009; Gatti et al., 2009; Clendenen et al., 2010; Rapchuk et al., 2010; Pesonen et al., 2011; Yucel et al., 2011; Chaparro et al., 2012; Jain et al., 2012; Pota et al., 2012; Yadeau et al., 2012; Mercadante et al., 2013; Paul et al., 2013; Clarke et al., 2015; Chen et al., 2016; Dou et al., 2017; Wang et al., 2017; Hah et al., 2018; Jones et al., 2019; Hermann et al., 2020; Jung et al., 2020; Teng et al., 2021) and 7 non-RCTs (Caraceni et al., 1999; Li et al., 2010; Savelloni et al., 2017; Peckham et al., 2018; Bykov et al., 2020; Chae et al., 2021; Dai et al., 2021); mortality was reported in 10 non-RCTs. (Abrahamsson et al., 2017; Gomes et al., 2017; Gomes et al., 2018; Slavova et al., 2018; Macleod et al., 2019; Lynn et al., 2020; Waddy et al., 2020; Bishop-Freeman et al., 2021; Mariottini et al., 2021; Chen et al., 2022).

### 3.2 Study characteristics

Table 1 summarizes the characteristics of the 26 RCTs (Caraceni et al., 2004; Gilron et al., 2005; Fassoulaki et al., 2006; Turan et al., 2006; Keskinbora et al., 2007; Clarke et al., 2009; Gatti et al., 2009; Clendenen et al., 2010; Rapchuk et al., 2010; Pesonen et al., 2011; Yucel et al., 2011; Chaparro et al., 2012; Jain et al., 2012; Pota et al., 2012; Yadeau et al., 2012; Mercadante et al., 2013; Paul et al., 2013; Clarke et al., 2015; Chen et al., 2016; Dou et al., 2017; Wang et al., 2017; Hah et al., 2018; Jones et al., 2019; Hermann et al., 2020; Jung et al., 2020; Teng et al., 2021) and 7 non-RCTs (Caraceni et al., 1999; Li et al., 2010; Savelloni et al., 2017; Peckham et al., 2018; Bykov et al., 2020; Chae et al., 2021; Dai et al., 2021) reporting TRAE risks. In the RCTs, the number of participants ranged from 29 to 410 per study, totaling 2,335 participants. The mean age of the participants in each study ranged between 34.1 and 79.6 years. The indications included perioperative pain (13 studies), (Fassoulaki et al., 2006; Turan et al., 2006; Clarke et al., 2009; Clendenen et al., 2010; Rapchuk et al., 2010; Pesonen et al., 2011; Yucel et al., 2011; Chaparro et al., 2012; Jain et al., 2012; Yadeau et al., 2012; Paul et al., 2013; Clarke et al., 2015; Hah et al., 2018) cancer-related pain (7 studies), (Caraceni et al., 2004; Keskinbora et al., 2007; Mercadante et al., 2013; Chen et al., 2016; Dou et al., 2017; Hermann et al., 2020; Teng et al., 2021) and non-cancer chronic pain (6 studies) (Gilron et al., 2005; Gatti et al., 2009; Pota et al., 2012; Wang et al., 2017; Jones et al., 2019; Jung et al., 2020).

**TABLE 1:** Characteristics of studies reporting the risk of central nervous system depression and gastrointestinal disorders.

Study, year (country)	Study medications					Main indication	Participants				Safety outcomes
	Opioid (route)	Opioid oral MME, mean ± SD	Gabapentinoids	Gabapentinoid dose, mg/day	Overlap days		Study groups	N	Male, %	Age, years, mean ± SD	
***RCTs; Perioperative pain***											
Fassoulaki et al. (2006) (United States)	Morphine (inj)	85.2 ± 36.3^d^; 105.0 ± 47.1^d^	Gabapentin	2,400	5D	Abdominal hysterectomy^a^	Opi + GABA; Opi	25; 27	0; 0	42 ± 5.6; 42 ± 6.2	CNS: sedation, dizziness; GI: N/V
Turan et al. (2006) (Turkey)	Fentanyl (inj)	NA^e^	Gabapentin	1,200; -	3D	Elective lower limb surgery^a^	Opi + GABA; Opi	20; 20	100; 100	54 (25–68)^f^; 50 (28–74)^f^	CNS: sedation, dizziness; GI: N/V, constipation
Clarke et al. (2009) (Canada)	Morphine (inj)	220.2 ± 86.4^d^; 162.0 ± 107.1^d^; 132.0 ± 60.0^d^; 285.6 ± 179.1^d^	Gabapentin	300; 600; 900; -	4D	Total knee arthroplasty^b^	Opi + GABA 300; Opi + GABA 600; Opi + GABA 900; Opi	7; 8; 7; 7	42.9; 50.0; 42.9; 28.6	60.7 ± 6.6; 57.3 ± 7.4; 65.8 ± 6.5; 62.33 ± 6.6	CNS: sedation, dizziness; GI: N/V
Clendenen et al. (2010) (United States)	Oxycodone (O)	76.5 ± 60^d^; 96 ± 63^d^	Pregabalin	300; -	2D	Arthroscopic rotator cuff repair of the shoulder^c^	Opi + GABA; Opi	23; 24	74.0; 79.0	63 ± 11; 60 ± 10	CNS: sedation, dizziness; GI: Nausea
Rapchuk et al. (2010) (Australia)	Fentanyl (inj)	271 ± 199^d^; 312.4 ± 211.2^d^	Gabapentin	1,200	2D	Cardiac surgery^a^	Opi + GABA; Opi	27; 27	81.5; 96.3	61.8 ± 8.7; 58.6 ± 11.1	CNS: sedation, dizziness
Pesonen et al. (2011) (Finland)	Oxycodone (inj, O)	72 ± 42^g^; 139.5 ± 66^g^	Pregabalin	150; -	5D	Cardiac surgery^a^	Opi + GABA; Opi	35; 35	60.0; 45.7	79.5 (75–89)^f^; 79.6 (75–91)^f^	CNS: sedation, cognitive dysfunction; GI: N/V
Yücel et al. (2011) (Turkey)	Morphine (inj)	101.4 ± 17.3^h^; 122.4 ± 10.2^h^; 140.9 ± 20.0^h^	Pregabalin	600; 300; -	1D	Hysterectomy^a^	Opi + GABA 600; Opi + GABA 300; Opi	30; 30; 30	0; 0; 0	43.3 ± 7.4; 46. 4 ± 9.1; 42.5 ± 9.3	CNS: dizziness; GI: N/V
Jain et al. (2012) (India)	Morphine (inj)	Day 2: 9.9 ± 3.3; Day 2: 1 8 ± 7.2	Pregabalin	150; -	2D	Total knee arthroplasty^b^	Opi + GABA; Opi	20; 20	45.0; 25.0	59.7 ± 8.8; 57.1 ± 8.8	CNS: dizziness; GI: N/V, constipation
Yadeau et al. (2012) (United States)	Hydromorphone (Inj), oxycodone/hydrocodone/hydromorphone (O)	Day 2: 70.4 ± 46.5; Day 2: 69.0 ± 62.7	Pregabalin	100; -	2D	Foot or ankle surgery^b^	Opi + GABA; Opi	28; 28	39.3; 35.7	60 ± 9; 61 ± 1	CNS: sedation, respiratory depression; GI: N/V, constipation
Chaparro et al. (2012) (Japan)	Morphine (inj), codeine, tramadol, hydrocodone (O)	Day 4: 0 (0–6)^i^; Day 4: 6 (0–12)^i^	Pregabalin	150; -	4D	Cosmetic surgery^a^	Opi + GABA; Opi	5; 49	0; 0	32.8 ± 8.7; 34.3 ± 9.8	CNS: sedation; GI: N/V
Paul et al. (2013) (Canada)	Morphine (inj)	198.9^d^ ^,^ ^j^; 217.5^d^ ^,j^	Gabapentin	600; -	2D	Total knee arthroplasty^b^	Opi + GABA; Opi	52; 49	36.5; 36.7	62.1 ± 6.4; 63.5 ± 6.7	CNS: sedation, dizziness, respiratory depression; GI: N/V
Clarke et al. (2015) (Canada)	Morphine (inj)	119.7 ± 85.2^h^; 162 ± 93.6^h^	Pregabalin	150	7D	Total hip arthroplasty^b^	Opi + GABA; Opi	83; 79	49.4; 51.9	60.2 ± 9.5 60.1 ± 8.8	CNS: sedation, dizziness; GI: N/V
Hah et al. (2018) (United States)	NA	NA	Gabapentin	1,800	3D	Surgeries^a^ ^,^ ^b^	Opi + GABA; Opi	208; 202	37.5; 43.1	57.0 ± 11.7; 56.4 ± 11.8	CNS: sedation, dizziness; GI: nausea
***RCTs; Cancer-associated pain***											
Caraceni et al. (2004) (Italy)	NA	116.5 ± 118.0/day; 106.6 ± 86.9/day	Gabapentin	600–1,800; -	10D	Neuropathic Cancer Pain	Opi + GABA; Opi	80; 41	43.8; 43.9	59.0 ± 11.0; 60.7 ± 11.0	CNS: sedation, respiratory depression
Keskinbora et al. (2007) (Turkey)	Tramadol (O), Fentanyl (P), Morphine (O)	NA^k^	Gabapentin	629.0 ± 303; -	2W	Neuropathic cancer pain	Opi + GABA; Opi	31; 32	71.0; 62.5	57.6 ± 14.8; 52.3 ± 16.3	CNS: sedation, dizziness; GI: N/V, constipation
Mercadante et al. (2013) (Italy)	Morphine (O)	85.7 ± 51.2/day; 75.4 ± 18.9/day	Pregabalin	119.2 ± 43.4; -	8W	Cancer pain	Opi + GABA; Opi	28; 16	NA; NA	65.5 ± 10.3	CNS: dizziness, cognitive dysfunction; GI: N/V, constipation
Chen et al. (2016) (China)	Oxycodone + prn morphine (O)	76.1 ± 17.1/day; 109.1 ± 27.9/day	Gabapentin	NA	6M	NA	Opi + GABA; Opi	30; 30	56.7; 63.3	65 ± 6; 67 ± 6	CNS: sedation, dizziness; GI: N/V, constipation
Dou et al. (2017) (China)	Morphine (O)	184.4 ± 69.9/day; 228.7 ± 66.9/day	Pregabalin	150; -	4W	Neuropathic cancer pain	Opi + GABA; Opi	40	60.0	33–80^l^	CNS: sedation, dizziness; GI: N/V
Hermann et al. (2020) (United States)	Fentanyl (P) + hydrocodone (O); Methadone (O) + oxycodone (O)	NA	Gabapentin	2,700; 900	4W	Chemoradiation for head and neck squamous cell cancer	Opi + GABA 2,700; Opi + GABA 900	31; 29	87.1; 93.1	61 (47–75)^m^; 60 (42–77)^m^	GI: N/V, constipation
Teng et al. (2021) (China)	Morphine (inj)	10.6 ± 3.9/day; 13.9 ± 3.8/day	Gabapentin	900	3M	Cancer pain	Opi + GABA; Opi	34; 40	55.9; 57.5	59.0 ± 6.2; 57.1 ± 6.1	CNS: sedation, dizziness, cognitive dysfunction; GI: N/V
***RCTs; Non-cancer chronic pain***											
Gilron et al. (2005) (Canada)	Morphine (O)	34.4 ± 2.6/day^n^; 45.3 ± 3.9/day^n^	Gabapentin	1,705 ± 83^n^	1W	Diabetic neuropathy, Postherpetic neuralgia	Opi + GABA; Opi	41	NA	-	CNS: sedation. dizziness, cognitive dysfunction; GI: N/V
Gatti et al. (2009) (Italy)	Oxycodone (O)	53.7/day^j^; 69.15/day^j^	Pregabalin	141.5^j^	3M	Neuropathic pain	Opi + GABA; Opi	169; 106	45.0; 36.8	62 (21–84)^f^; 65 (37–90)^f^	CNS: sedation, dizziness; GI: N/V, constipation
Pota et al. (2012) (Italy)	Buprenorphine (P)	63/day^o^; 63/day^o^	Pregabalin	300	3W	Chronic back pain	Opi + GABA; Opi	22; 22	NA; NA	35–80^l^	CNS: sedation, dizziness; GI: nausea, constipation
Wang et al. (2017) (China)	Morphine (O)	41.8/day^j^; 52.8/day^j^	Pregabalin	142.5^j^	3M	Chronic neuropathic pain	Opi + GABA; Opi	128; 90	46.9; 46.7	18–89^k^	CNS: sedation, dizziness; GI: N/V, constipation
Jones et al. (2019) (United States)	Morphine (inj), hydromorphone (inj), oxycodone (O)	116.9 (80.7–207.5)/day^i^; 60.35 (4.6–148.0)/day^i^; 73.3 (0–141.3)/day^i^	Pregabalin	200; 300	18D	Burn injuries related pain	Opi + GABA 200; Opi + GABA 300; Opi	18; 14; 19	83.3; 64.3; 89.5	36 ± 11.4; 42.6 ± 14.1; 37.5 ± 12	CNS: dizziness; GI: nausea
Jung et al. (2020) (Korea)	Oxycodone (O)	22.5/day^o^	Pregabalin	600	8W	Cervical myelopathy, neuropathic pain	Opi + GABA; Opi	20; 19	55.0; 63.1	57.5 ± 12.7; 52.8 ± 11.4	CNS: sedation, dizziness; GI: nausea, constipation
***Non-randomized prospective study***											
Li et al. (2010) (China)	Oxycodone (O)	60.0 ± 35.6/day; 81.9 ± 32.8/day	Gabapentin	862.5 ± 282.6	2W	Malignant neuropathic pain	Opi + GABA; Opi	32; 21	56.3; 42.9	57.3 ± 13.2; 57.1 ± 12.4	CNS: sedation, dizziness; GI: N/V, constipation
***Retrospective studies***											
Savelloni et al. (2017) (United States)	NL	89.9 ± 115; 63.9 ± 70.5	Gabapentin; Pregabalin	NA^p^	NA	Opioids and naloxone user	Opi + GABA; Opi	36; 89	33.3; 51.7	NA^q^	CNS: sedation, respiratory depression
Peckham et al. (2018) (United States)	NL	NA	Gabapentin	NA	≥120D	Opi and/or GABA user^r^	Opi + GABA; Opi	15,343; 736,835	35.2^s^; 39.4^s^	50^s^ ^,^ ^t^; 44^s^ ^,t^	CNS: respiratory depression
Bykov et al. (2020) (United States)	NL	283.2 ± 357.2; 283.8 ± 356.7	Gabapentin; Pregabalin	NA	NA	Major surgeries^u^	Opi + GABA; Opi	892,484; 4,655,183	39.6; 41.1	63.6 ± 12.0; 63.6 ± 12.0	CNS: respiratory depression
Chae et al. (2021) (Korea)	Oxycodone (O), morphine (inj)	30.0 ± 30.6^v^; 40.8 ± 30.3^v^	Pregabalin	150-	2D	arthroscopic rotator cuff repair surgery	Opi + GABA; Opi	32; 32	43.8; 50.0	61.6 ± 8.9; 59.8 ± 9.0	CNS: dizziness; GI: N/V, constipation
Dai et al. (2021) (China)^48^	Morphine (O)	39.5 ± 16.0; 61.5 ± 19.3	Pregabalin	150-	NA	Pancreatic cancer	Opi + GABA; Opi	120; 120	56.7; 59.2	65 ± 8; 63 ± 6	CNS: sedation, dizziness, cognitive dysfunction; GI: N/V
Caraceni et al. (1999) (Italy)	NL	147 ± 228	Gabapentin	1,004 ± 262	2W	Neuropathic cancer pain	Opi + GABA; Opi	22	18.2	49.3 (16–77)^f^	CNS: sedation, dizziness, cognitive dysfunction; GI: N/V, constipation

Abbreviations: CNS, central nervous system; D, days; GABA, gabapentinoid; GI, gastrointestinal; Inj, injection; MME, morphine milligram equivalents; N, number; NA, not available; NL, not limited; N/V, nausea/vomiting; O, oral; Opi, opioid analgesics; P, patch; RCTs, randomized controlled trial; SD, standard deviation; W, weeks.

a General anesthesia.

b Spinal-epidural anesthetic.

c Interscalene brachial plexus block.

d Cumulative morphine consumption for 48 h.

e Number of patient-controlled analgesics including fentanyl at postoperative 48–72 h; Opi + GABA 2 ± 3; Opi alone 8 ± 5

f Mean (range).

g Mean cumulative total oxycodone consumption from extubation (< 24 h after operation) to the end of the 5th day (IV, oral).

h Cumulative morphine consumption for the overlap period.

i Median (IQR).

j Mean daily doses at the end.

k Baseline mean opioid consumption on the day of randomization [each opioid analgesic, Opi + GABA group vs. Opi group]; oral tramadol (MME/day):40 ± 0 (14 patients) vs. 40 ± (22 patients), fentanyl patch (MME/48 h):81.8 ± 55.5/48 h (11 patients) vs. 120 ± 60/48 h (3 patients); morphine sustained release (MME/day):90 ± 60 (6 patients) vs. 65 ± 44.2 (7 patients).

l Age range for all participants.

m Median (range).

n Mean ± standard error.

o Fixed-dose.

p High dose group for gabapentinoids: total daily doses of gabapentin ≥ 1,800 mg or pregabalin ≥300 mg.

q The proportion of elderly patients (≥ 65 years old) in each group: Opi + GABA group 33.3% vs. Opi only group 55%.

r At least 120 days of opioid and/or gabapentinoid use during the 12-month cohort identification period.

s Among three cohorts, the value for the non-overuse group, which was the largest group.

t Mean.

u Major surgery for hip or knee arthroplasty, coronary artery bypass grafting, cholecystectomy, colorectal resection, cystectomy, esophagectomy, gastrectomy, hysterectomy, laminectomy or spinal fusion, lobectomy, mastectomy, nephrectomy, pancreatectomy, or surgery for hip fracture or dislocation.

v MME of IV morphine during two days after the operation.

In non-RCTs, the number of participants ranged from 22 to 5,547,667 per study, totaling 6,300,349 participants, with a mean age of 44.4–64.0 years. The indications included perioperative pain (2 studies), (Bykov et al., 2020; Chae et al., 2021) cancer-related pain (3 studies), (Caraceni et al., 1999; Li et al., 2010; Dai et al., 2021) and non-cancer chronic pain (2 studies), (Savelloni et al., 2017; Peckham et al., 2018).

Only ten non-RCTs reported mortality (Table 2) (Abrahamsson et al., 2017; Gomes et al., 2017; Gomes et al., 2018; Slavova et al., 2018; Macleod et al., 2019; Lynn et al., 2020; Waddy et al., 2020; Bishop-Freeman et al., 2021; Mariottini et al., 2021; Chen et al., 2022). Six studies using health databases included 226,940 patients with a mean age of 38.8–49.3 years and a follow-up period of 3–20 years (Abrahamsson et al., 2017; Gomes et al., 2017; Gomes et al., 2018; Macleod et al., 2019; Waddy et al., 2020; Chen et al., 2022). Four other studies using post-mortem databases on poisoning-related deaths involved 6,581 patients (Slavova et al., 2018; Lynn et al., 2020; Bishop-Freeman et al., 2021; Mariottini et al., 2021).

**TABLE 2:** Characteristics of studies reporting mortality risk.

Study, year; (country)	Study period	Study medications			Study population	Participants				Details
		Opioid (route)	Gabapentinoid	Overlap definition		Group definition	N	Male, %	Age, year, mean ± SD	
***Case-control studies***										
Gomes et al. (2017) (Canada)	1997–2013	Prescribed opioids (non-parenteral)	Gabapentin	within 120 days preceding death	Non-parenteral opioid user for non-cancer pain	Case: opioid-related death; Control: matched using a disease risk index	1,256; 4,619	57.0; 56.7	47.5 ± 10.0; 47.8 ± 9.9	opioid-related death confirmed by the investigating coroner, excluding suicides or homicides
Gomes et al. (2018) (Canada)	1997–2016	Prescribed opioids (non-parenteral)	Pregabalin	within 120 days preceding death	Non-parenteral opioid user for non-cancer pain	Case: opioid-related death Control: matched using a disease risk index	1,417; 5,097	56.5; 55.3	48 (42–54)^a^; 49 (42–54)^a^	opioid-related death confirmed by the investigating coroner, excluding suicides or homicides
Chen et al. (2022) (United Kingdom)	2000–2015	Prescribed opioids (non-parenteral)	Gabapentin, pregabalin	NA	Non-parenteral opioid user with a minimum 1-year follow-up period	Case: opioid-related death; Control: matched using a disease risk score	230; 920	53.5; 53.9	50.1 ± 14.1; 49.1 ± 14.3	opioid-related death: ICD-10 code; F11–F16, F18–F19, X40–X44, X60–X64, X85, Y10–Y14
***Retrospective cohort studies***										
Abrahamsson et al. (2017) (Sweden)	2005–2012	buprenorphine, methadone	Pregabalin	NA	Patients with OST	Total; Deceased	4,501; 356	73.8; 80.1	34.4 (28.7–42.1)^a^; 38.7 (29.9–44.9)^a^	all-cause death; non-overdose death; overdose death: ICD-10 code; X40-49 or Y10-19
MacLeod et al. (2019) (United Kingdom)	1998–2014	Buprenorphine, methadone	Gabapentin, pregabalin	prescribed during OST and op to 12 months post-treatment	Patients with OST	Total; Deceased	12,118; 7,106	67.3; 68.1	38.8 ± 10.4; 39.3 ± 10.7	all-cause death; non-drug-related poisoning; non-drug-related poisoning-F11–F16, F18–F19, X40–X44, X85, Y10–Y14
Waddy et al. (2020) (United States)	2010–2012	Prescribed opioids (non-parenteral)	Gabapentin; pregabalin	NA	Patients with ESRD	Opi + GABA; Opi	28,153; 168,629	44.3; 49.6	≥20^b^	2-year all-cause death
***Cross-sectional studies; post-mortem data***										
Slavova et al. (2018) (United States)	2015	Not limited	gabapentin	identified in sample	All poisoning death	Total	4,169	60.9	NA	GABA+/Opi+: 876 (21.0%); GABA+/Opi-: 55 (1.3%); GABA-/Opi+: 2,479 (59.5%); GABA-/Opi-: 759 (18.2%)
Lynn et al. (2020) (Ireland)	2013–2016	Not limited	Pregabalin	NA	All poisoning death	Total	1,489	NA	NA	GABA +/Opi+: 211 (14.2%); GABA+/Opi-: 29 (1.9%); GABA-/Opi+: 658 (44.2%); GABA-/Opi-: 591 (39.7%)
Mariottini et al. (2021) (Finland)	2016–2019	Including buprenorphine	Gabapentin, pregabalin	NA	Poisoning death with buprenorphine or norbuprenorphine finding from a sample	Total	792	85.6	34 (26–43)^a^	GABA+: 349 (44.1%); GABA-: 443 (55.9%)
Bishop-Freeman et al. (2021) (United States)	2010–2018	Including buprenorphine	Gabapentin, pregabalin	NA	Poisoning death with buprenorphine finding from a sample	Total	131	64.1	14–64^c^	GABA+: 38 (29.0%); GABA-: 93 (71.0%)

AbbreviationsGABA, gabapentinoid; NA, not available; ESRD, end-stage renal disease; ICD, international classification of diseases; N, number; NA, not available; OST, opioid substitution therapy; Opi, opioids; SD, standard deviation.

a Median (interquartile range).

b Adult patients ≥ 20 years old.

c Range.

### 3.3 Treatment-related adverse events

**FIGURE 1:**
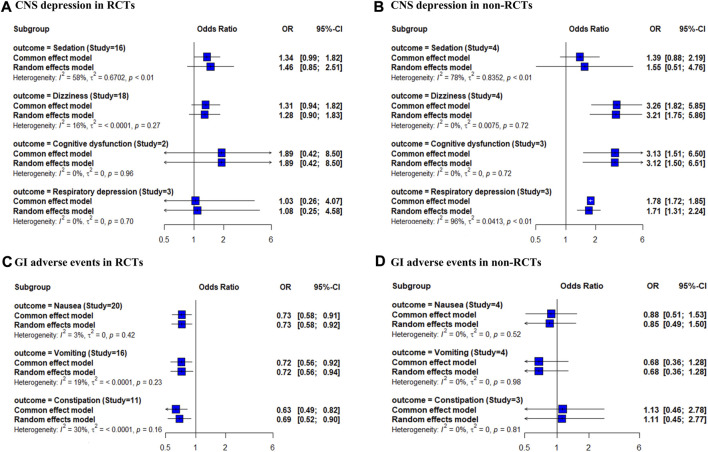
Forest plot of the risk of CNS depression and GI adverse events in opioid and gabapentinoid combination therapy compared with opioid therapy. **(A)** CNS depression in RCTs, **(B)** CNS depression in non-RCTs, **(C)** GI adverse events in RCTs, and **(D)** GI adverse events in non-RCTs. CNS, central nervous system; GI, gastrointestinal; RCTs, randomized controlled trials.

The risks of sedation, dizziness, cognitive dysfunction, and respiratory depression were reported in 16, 18, 2, and 3 RCTs, and 4, 4, 3, and 3 non-RCTs, respectively. The risks of nausea, vomiting, and constipation were reported in 20, 16, and 11 RCTs and 4, 4, and 3 non-RCTs, respectively. In the RCT-only analysis, the risk of sedation, dizziness, and cognitive dysfunction showed an increasing trend for combination therapy compared with that for opioid therapy; however, the differences were not significant (Figure 1). In the non-RCT-only analysis, the use of combination therapy was significantly associated with an increased risk of dizziness, cognitive dysfunction, and respiratory depression (OR 3.26, 95% CI 1.82–5.85; OR 3.13, 95% CI 1.51–6.50; OR 1.71, 95% CI 1.31–2.24, respectively). The risks of nausea, vomiting, and constipation were significantly decreased in combination therapy compared to opioid therapy in the RCT-only analysis (OR 0.73, 95% CI 0.58–0.91; OR 0.72, 95% CI 0.56–0.92; OR 0.63, 95% CI 0.49–0.82, respectively). None of the GI adverse events were significantly different between combination therapy and opioid therapy in the non-RCT-only analysis. Forest plots of individual studies and pooled estimates of the risks of CNS depression and GI adverse events are presented in Supplementary Figures S2, S3, respectively.

The results of the subgroup and meta-regression analyses in RCTs revealed significant differences among the indications in the risk of sedation and constipation (*p* < 0.01, Table 3). Combination therapy for cancer pain was associated with the highest risk of sedation (OR 3.45, 95% CI 1.93–6.18) and the lowest risk of constipation (OR 0.04, 95% CI 0.01–0.25). In the subgroup analysis of the risk of nausea and vomiting, perioperative pain, a treatment period of ≤ 7days, and an opioid dose ≥ 50 MME/day showed a significantly decreased risk.

**TABLE 3:** Subgroup and meta-regression analyses of the risk of central nervous system depression and gastrointestinal disorders in randomized controlled trials.

Variables	Number of studies; (sample size)	Subgroup analysis			Meta-regression analysis	
		Pooled ORs (95% CI)	I^2^ (%)	*p*-value^*^	Beta coefficients (95% CI)	*p*-value
***Sedation***						
Indications						
Perioperative	6 (377)	1.41 (0.88–2.26)	14	< 0.01	Ref	
Non-cancer chronic	4 (558)	0.34 (0.17–0.67)	37		−1.37 (−2.24‒0.49)	< 0.01
Cancer	6 (445)	3.45 (1.93–6.18)	0		0.89 (0.12–1.67)	0.02
Duration of combination therapy						
≤ 7 days	7 (398)	1.41 (0.89–2.24)	0	0.86	Ref	
< 7 days	9 (982)	1.40 (0.66–3.00)	73		−0.10 (−1.22‒1.02)	0.86
Opioid dose						
< 50 MME/day	3 (194)	1.95 (0.95–4.02)	0	0.60	Ref	
≥ 50 MME/day	9 (818)	1.31 (0.65–2.64)	64		−0.37 (−1.74–1.01)	0.60
No difference in opioid dose between groups	8 (549)	1.45 (0.95–2.22)	9	-	-	-
Gabapentinoid dose						
< 1 DDD/day	12 (1,208)	1.30 (0.70–2.43)	63	0.65	Ref	
≥ 1 DDD/day	3 (112)	1.97 (0.84–4.61)	40		0.34 (−1.12‒1.81)	0.65
***Dizziness***						
Indications						
Perioperative	7 (408)	1.23 (0.80–1.91)	38	0.07	Ref	
Non-cancer chronic	6 (650)	0.56 (0.22–1.45)	0		−0.64 (−1.84‒0.57)	0.30
Cancer	5 (397)	2.13 (1.14–3.98)	0		0.52 (−0.28‒1.31)	0.20
Duration of combination therapy						
≤7 days	8 (431)	1.24 (0.81–1.91)	28	0.71	Ref	
>7 days	10 (1,024)	1.41 (0.84–2.37)	11		0.21 (−0.52‒0.94)	0.57
Opioid dose						
< 50 MME/day	4 (176)	2.20 (0.89–5.45)	0	0.17	Ref	
≥ 50 MME/day	10 (911)	1.10 (0.74–1.64)	30		-0.68 (-1.69‒0.34)	0.19
No difference in opioid dose between groups	8 (492)	1.00 (0.62–1.60)	18	-	-	-
Gabapentinoid dose					-	-
< 1 DDD/day	12 (1,152)	1.35 (0.90–2.02)	37	0.73	Ref	
≥ 1 DDD/day	5 (243)	1.18 (0.60–2.29)	0		−0.01 (−0.88‒0.85)	0.97
***Respiratory depression***						
Indications						
Perioperative	2 (155)	0.93 (0.20–4.31)	0	0.77	Ref	
Non-cancer chronic	0 (0)	-	-		-	
Cancer	1 (120)	1.59 (0.06–39.80)	-		0.49 (−3.12‒4.09)	0.79
Duration of combination therapy						
≤ 7 days	2 (155)	0.93 (0.20–4.31)	0	0.77	Ref	
> 7 days	1 (120)	1.59 (0.06–39.80)			0.49 (−3.12‒4.09)	0.79
Opioid dose						
< 50 MME/day	0 (0)	-			-	-
≥ 50 MME/day	3 (275)	1.03 (0.26–4.07)	0		-	-
No difference in opioid dose between groups	3 (275)	1.03 (0.26–4.07)	0	-	-	-
Gabapentinoid dose						
< 1 DDD/day	0 (0)	-			-	-
≥ 1 DDD/day	3 (275)	1.03 (0.26–4.07)	0		-	-
***Nausea***						
Indications						
Perioperative	10 (987)	0.72 (0.56–0.94)	15	1.00	Ref	
Non-cancer chronic	5 (430)	0.72 (0.33–1.56)	0		0.04 (−0.85‒0.92)	0.94
Cancer	5 (414)	0.74 (0.43–1.28)	49		0.04 (−0.66‒0.75)	0.91
Duration of combination therapy						
≤ 7 days	11 (1,008)	0.72 (0.56–0.94)	6	0.98	Ref	
> 7 days	9 (823)	0.73 (0.47–1.15)	10		0.02 (-0.55‒0.59)	0.95
Opioid dose						
< 50 MME/day	6 (343)	0.90 (0.56–1.44)	0	0.18	Ref	
≥ 50 MME/day	10 (928)	0.59 (0.40–0.87)	0		−0.47 (−1.10‒0.16)	0.14
No difference in opioid dose between groups	9 (608)	0.83 (0.55–1.26)	14	-	-	-
Gabapentinoid dose						
< 1 DDD/day	13 (1,120)	0.71 (0.52–0.98)	13	0.61	Ref	
≥ 1 DDD/day	6 (651)	0.81 (0.57–1.14)	0		0.12 (−0.37‒0.61)	0.62
***Vomiting***						
Indications						
Perioperative	9 (940)	0.70 (0.52–0.94)	19	0.86	Ref	
Non-cancer chronic	2 (296)	0.91 (0.35–2.34)	0		0.30 (−0.79‒1.40)	0.58
Cancer	5 (397)	0.73 (0.38–1.39)	51		0.07 (−0.67‒0.82)	0.85
Duration of combination therapy						
≤ 7 days	10 (961)	0.71 (0.53–0.95)	14	0.81	Ref	
> 7 days	6 (672)	0.76 (0.47–1.23)	39		0.05 (−0.57‒0.67)	0.88
Opioid dose						
< 50 MME/day	5 (304)	0.85 (0.51–1.40)	41	0.33	Ref	
≥ 50 MME/day	8 (816)	0.61 (0.41–0.93)	9		−0.44 (−1.11‒0.24)	0.20
No difference in opioid dose between groups	5 (410)	0.72 (0.43–1.20)	17	-	-	-
Gabapentinoid dose						
< 1 DDD/day	12 (1,052)	0.74 (0.53–1.03)	26	0.83	Ref	
≥ 1 DDD/day	3 (521)	0.78 (0.52–1.18)	0		0.04 (−0.50‒0.58)	0.89
***Constipation***						
Indications						
Perioperative	4 (544)	0.61 (0.42–0.89)	0	< 0.01	Ref	
Non-cancer chronic	5 (597)	0.92 (0.61–1.38)	0		0.41 (−0.14‒0.96)	0.15
Cancer	2 (124)	0.04 (0.01–0.25)	0		-2.64 (-4.40‒0.89)	< 0.01
Duration of combination therapy						
≤7 days	5 (565)	0.62 (0.43–0.89)	0	0.41	Ref	
>7 days	6 (700)	0.78 (0.52–1.17)	61		0.23 (−0.32‒0.77)	0.41
Opioid dose						
<50 MME/day	3 (100)	0.81 (0.28–2.36)	0	0.78	Ref	
≥50 MME/day	4 (433)	0.64 (0.27–1.50)	67		−0.22 (−1.80‒1.36)	0.78
No difference in opioid dose between groups	3 (121)	0.38 (0.09–1.68)	70		-	-
Gabapentinoid dose						
<1 DDD/day	6 (691)	0.74 (0.51–1.08)	0	0.62	Ref	
≥1 DDD/day	4 (514)	0.65 (0.44–0.96)	0		−0.21 (−0.76‒0.34)	0.45

^*^ *p*-value for subgroup differences.

Note: Significance level < 0.05 (in bold**)**.

Abbreviations: CI, confidence interval; DDD, defined daily dose; MME, morphine milligram equivalents; ORs, odds ratio; ref, reference.

### 3.4 Mortality risk

**FIGURE 2:**
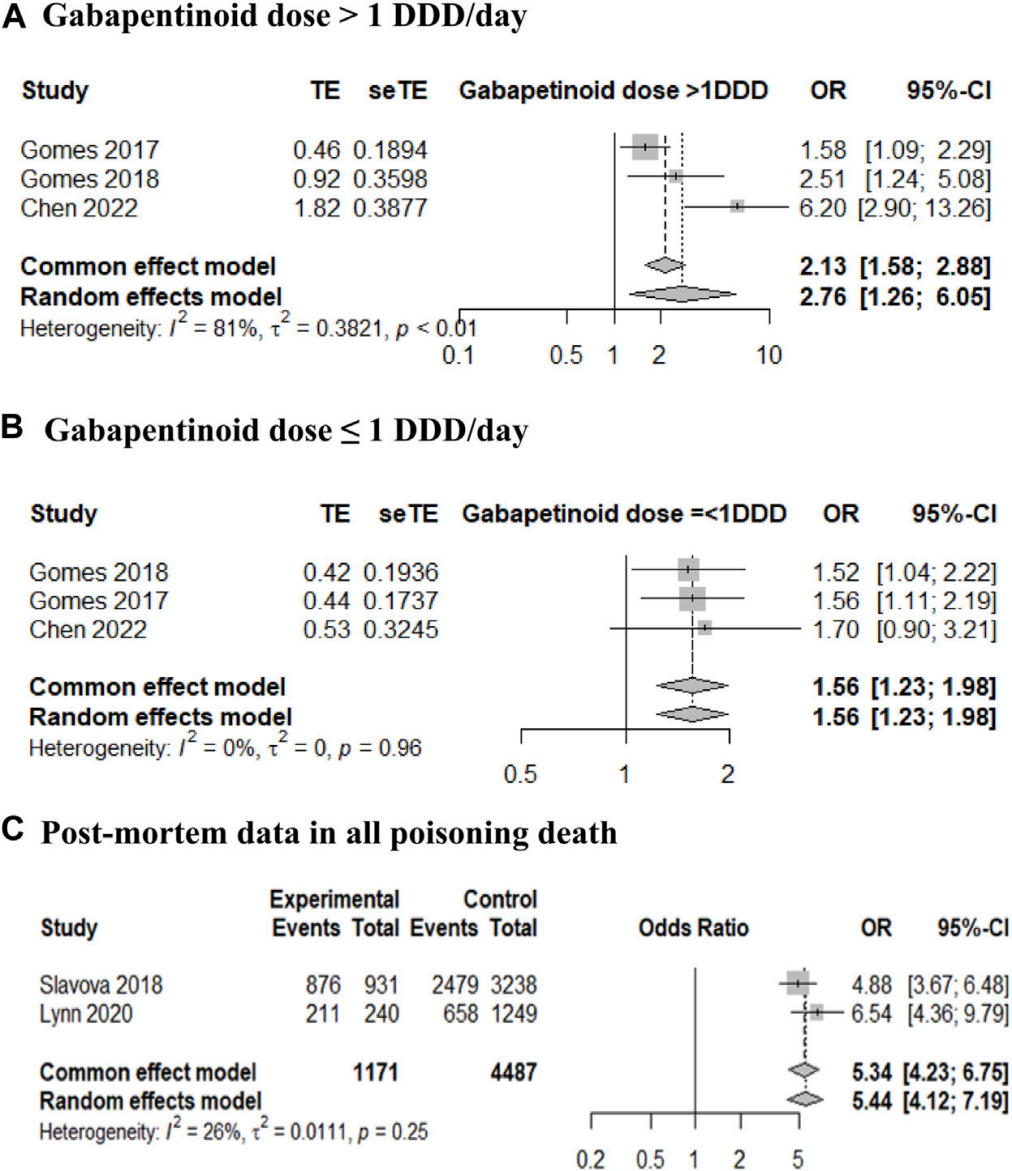
Forest plot of the mortality risk in opioid and gabapentinoid combination therapy compared with that in opioid therapy. **(A)** Gabapentinoid dose > 1 defined daily dose (DDD)/day, **(B)** gabapentinoid dose ≤ 1 DDD/day, and **(C)** post-mortem data in all poisoning deaths.

Three case-control studies were analyzed according to gabapentinoid dose, as presented in the included studies (Gomes et al., 2017; Gomes et al., 2018; Chen et al., 2022). For gabapentinoid dose > 1 DDD/day, the adjusted mortality OR was 2.76 with a 95% CI of 1.26–6.05 (Figure 2A), and for gabapentinoid dose ≤ 1 DDD/day, the adjusted mortality OR was 1.56 with a 95% CI of 1.23–1.98 (Figure 2B). The adjusted HR of mortality was also estimated from the data of two retrospective cohort studies (Abrahamsson et al., 2017; Macleod et al., 2019), and showed a similar trend (adjusted HR 1.73, 95% CI 1.38–2.17; data not shown). One cohort study was excluded from the meta-analysis because the reference group did not receive opioid therapy (reference group; without use of either opioid or gabapentinoid). The mortality HR of the combination therapy group was greater than that of the opioid therapy group as follows: gabapentin model, 1.16 (1.12–1.19) and 1.12 (1.09–1.15) and pregabalin model, 1.22 (1.16–1.28) and 1.12 (1.09–1.14) (Waddy et al., 2020).

When analyzing two post-mortem cross-sectional studies of all deaths from poisoning (Slavova et al., 2018; Lynn et al., 2020), the OR for gabapentinoid identification in opioid users was 5.34 with a 95% CI of 4.23–6.75 (Figure 2C). In two studies that included deaths due to poisoning with buprenorphine findings (Bishop-Freeman et al., 2021; Mariottini et al., 2021), the prevalence of gabapentinoid combination (29.0% and 44.1%) was similar to that reported in studies of all poisoning deaths (24.3% and 26.1%, respectively).

### 3.5 Risk of bias, publication bias, and sensitivity analysis

Approximately two-thirds of the 26 RCTs (34.6%) were of some concern or had a high risk of bias (Supplementary Table S3). Among the 13 non-RCTs, over three-quarters had a serious risk of bias (Supplementary Table S4). Visual inspection of the funnel plot and Egger’s test revealed no publication bias (Supplementary Figure S4).

The results of the sensitivity analysis of the RCT study quality are presented in Supplementary Table S5. When analyzing studies without a high or serious risk of bias, the results were similar to the overall findings. Notably, the risk of sedation and dizziness significantly increased with combination therapy only when superior quality RCTs were included. Sensitivity analysis showed no effect of the sample size on the risk of TRAEs (Supplementary Figure S5).

## 4 Discussion

This meta-analysis evaluated the safety of opioid and gabapentinoid combination therapy compared with that of opioid therapy. In the non-RCT analysis, combination therapy was significantly associated with an increased risk of dizziness, cognitive dysfunction, and respiratory depression. The risk of sedation in combination therapy in cancer patients was greater than that in other indications in the RCT subgroup analysis. The mortality risk associated with combination therapy was also higher than that with opioid therapy. Meanwhile, combination therapy was significantly associated with a decreased risk of GI adverse events in the RCT analysis.

The risk of CNS depression and death has been a major concern when opioid and gabapentinoid combination therapy is used in the elderly population (AGS Beers Criteria Update Expert Panel, 2019). Although it was not possible to conduct subgroup analysis based on age due to the wide range of ages in each study, CNS depression risk and death did not seem to be limited to elderly patients considering the age range in the included studies. This finding agrees with Bykov et al., who reported that the risk of opioid overdose in opioid and gabapentinoid combination therapy did not differ according to age (Bykov et al., 2020).

The increased risk of respiratory depression and mortality with the concurrent use of a gabapentinoid with an opioid could be explained by pharmacokinetic and pharmacodynamic interactions. The bioavailability of gabapentinoids is increased by opioids, which reduce intestinal motility (Eckhardt et al., 2000). Furthermore, gabapentinoids can reduce CO_2_ responsiveness in the medullary respiratory center in addition to the respiratory depressant effect of opioid analgesics (Henson and Ward, 1994; Becker and Haas, 2011). One animal study reported that a low dose of pregabalin could reverse tolerance to morphine respiratory depression, and a high dose of pregabalin alone could depress respiration (Lyndon et al., 2017). In addition, we could consider the abuse or misuse of gabapentinoids when interpreting mortality risk in combination therapy. Opioid-related and all-cause death is known to be associated with gabapentinoid abuse or misuse in patients undergoing opioid therapy, and opioid use disorder is one of the risk factors for gabapentinoid abuse or misuse (Hägg et al., 2020; Evoy et al., 2021). The results of studies on poisoning deaths included in this study could provide evidence for this aspect. In a similar context, more than two-thirds of deaths due to gabapentinoid poisoning were co-identified with opioids, and the association of gabapentinoid with poisoning-related deaths has been shown to increase (Häkkinen et al., 2014; Elliott et al., 2017; Faryar et al., 2019; Darke et al., 2021). The difference in the risk of respiratory depression between RCTs and non-RCTs might also be associated with the gabapentinoid use patterns in the real world. Therefore, when evaluating gabapentinoid use in patients, especially opioid users, healthcare professionals should consider these factors.

According to the subgroup analysis and meta-regression, the risks of sedation and dizziness with combination therapy were significantly higher in patients with cancer pain than in those with other indications. The risk of dizziness was also significantly increased in patients with cancer pain when the combination therapy was used. This might be because chemotherapy in cancer patients can damage progenitor cells and myelines (Clouston et al., 1992; Meyers, 2008). Close monitoring for sedation and dizziness is necessary for patients with advanced cancer when opioid and gabapentin combination therapy is used.

We confirmed a reduced risk of GI adverse events with combination therapy, especially in short-term (≤ 7 days) therapy, opioid doses of ≥ 50 MME/day, and perioperative pain. This could be explained by the opioid-sparing effects and tolerance development for GI adverse events of opioids (Kim et al., 2017). A short-term addition of gabapentinoids to high doses of opioids after surgery may be recommended to reduce nausea and vomiting.

Most previous systematic reviews and meta-analyses have focused on the perioperative use of gabapentinoids (Liu et al., 2017; Verret et al., 2020; Campbell et al., 2021). We evaluated the risk of opioid and gabapentinoid combination for any type of pain. To the best of our knowledge, ours is the first meta-analysis to evaluate the risk of two common adverse events, CNS depression and GI adverse events, of a combination of opioids and gabapentinoids, and to analyze data using RCTs and non-RCTs. We found that the risks of sedation, dizziness, and GI adverse events were typically assessed with RCTs, whereas the risks of cognitive disorder and respiratory depression were typically assessed with non-RCTs in a large patient population. We evaluated the pooled effect of the combination therapy on mortality in several ways. In RCT studies, CNS depression showed an increasing trend in combination therapy, and in non-RCT studies, although there was a serious risk of bias in over three-quarters of studies, the risk of dizziness, cognitive dysfunction, respiratory depression, and mortality showed a significant increase in combination therapy. The risk of CNS depression and mortality in combination therapy should be interpreted cautiously and confirmed through well-organized non-RCT or long-term RCT studies in the future.

Our study had several limitations. First, the studies included in the meta-analysis were heterogeneous in terms of the baseline characteristics of the population and overlap period, which may have influenced the results of the meta-analysis. To address this limitation, we performed subgroup analyses based on these factors. Second, approximately half of the included RCTs and most non-RCTs had an excessively high risk of bias. However, our sensitivity analyses, which only included studies with a low or moderate risk of bias, support the robustness and validity of our main findings. Third, the number of studies included in the analysis of cognitive dysfunction and respiratory depression is small. Additionally, the validity of findings for respiratory depression could be limited owing to the following factors: 1) the significance and effect size in the non-RCT analysis tended to depend on two retrospective studies, Bykov et al. and Peckham et al.; and 2) no differences in the risk of respiratory depression were identified in the RCT-only analysis. Lastly, the interpretation of mortality risk in combination therapy was limited owing to the absence of RCT or prospective studies with this aim. Therefore, studies providing a high level of evidence such as RCT or prospective studies are needed to confirm the risk of mortality.

In conclusion, combination therapy with opioids and gabapentinoids is associated with an increased risk of CNS depression and mortality, and a reduced risk of GI adverse events. However, caution is needed when interpreting the clinical meanings because no differences in the risk of respiratory depression were identified in the RCT-only analysis, and no RCT or prospective studies investigated mortality. Our data suggest that clinicians should be aware of these potential risks in adults, including the elderly, when combination therapy is initiated. Close monitoring of treatment-related adverse events is required during combination therapy, especially in patients with cancer, owing to an increased risk of CNS depression. Further research on drug safety is needed to establish practical evidence of the tolerability of combination therapies with opioids and gabapentinoids.

## Data Availability

The original contributions presented in the study are included in the article/Supplementary Material, further inquiries can be directed to the corresponding authors.

## Abbreviations

CI, confidence intervals; CNS, central nervous system; DDs, defined daily doses; GI, gastrointestinal; HR, hazard ratio; MME, morphine milligram equivalents; ORs, odds ratios; RCTs, randomized controlled trials; RoB 2, rish of bias 2; ROBINS-I, Cochrane Collaboration’s Risk of Bias in non-RCTs; TRAEs, treatment-related adverse events.
